# Antineoplastic Activity of Sodium Caseinate in a Cytarabine-Resistant Mouse Acute Myeloid Leukemia Cell Line

**DOI:** 10.3390/nu16183190

**Published:** 2024-09-20

**Authors:** Itzen Aguiñiga-Sánchez, Edgar Ledesma-Martínez, Mariana Vázquez-Guerrero, David Hernández-Álvarez, Amanda Velasco-García, Katia Michell Rodríguez-Terán, Daniel Romero-Trejo, Víctor Manuel Mendoza-Núñez, Víctor Manuel Macías-Zaragoza, Edelmiro Santiago-Osorio

**Affiliations:** 1 Hematopoiesis and Leukemia Laboratory, Research Unit on Cell Differentiation and Cancer, Faculty of High Studies Zaragoza, National Autonomous University of Mexico, Mexico City 09230, Mexico; 2 Department of Biomedical Sciences, School of Medicine, Faculty of High Studies Zaragoza, National Autonomous University of Mexico, Mexico City 56410, Mexico; 3 Research Unit on Gerontology, Faculty of High Studies Zaragoza, National Autonomous University of Mexico, Mexico City 09230, Mexico

**Keywords:** acute myeloid leukemia, chemoresistance, sodium caseinate, milk protein, apoptosis

## Abstract

Background: Acute myeloid leukemia (AML) is a hematological neoplasm of rapid and progressive onset, and is the most common form of leukemia in adults. Chemoresistance to conventional treatments such as cytarabine (Ara-C) and daunorubicin is a main cause of relapse, recurrence, metastasis, and high mortality in AML patients. It is known that sodium caseinate (SC), a salt derived from casein, a milk protein, inhibits growth and induces apoptosis in acute myeloid leukemia cells but not in normal hematopoietic cells. However, it is unknown whether SC retains its antileukemic effect in cytarabine-resistant AML cell lines. Objective: To evaluate the antineoplastic effect of SC in cytarabine-resistant leukemia models. Methods: The SC inhibits the growth and induces apoptosis in parental WEHI-3 AML cells. Here, we generated two cytarabine-resistant sublines, WEHI-CR25 and WEHI-CR50, which exhibit 6- and 16-fold increased resistance to cytarabine, respectively, compared to the parental WEHI-3 cells. Thus, these sublines mimic a chemoresistant model. Results: We demonstrate that WEHI-CR25 and WEHI-CR50 cells retain sensitivity to SC, similar to parental WEHI-3 cells. This sensitivity results in inhibited cell proliferation, induced apoptosis, and increased expression of ENT1 and dCK, molecules involved in the entry and metabolism of Ara-C, while decreasing MDR1 expression. Additionally, we observed that SC prolonged the survival of WEHI-CR50 tumor-bearing mice, despite their resistance to Ara-C. Conclusion: This is the first evidence that SC, a milk protein, may inhibit proliferation and induce apoptosis in cytarabine-resistant cells.

## 1. Introduction

Acute myeloid leukemia (AML) is a heterogeneous group of primary hematopoietic neoplasms arising from the aberrant proliferation and differentiation of hematopoietic progenitor cells [1]. AML is more prevalent in adults, and accounts for approximately 1% of all cancers [2]. Advances in the understanding of cancer molecular biology and the identification of mutations in AML have created favorable conditions for improving clinical outcomes [3]. In 2022, the International Consensus Classification (ICC) for myeloid and lymphoid neoplasms was published, aiming to better integrate genomic information, define new disease entities, and refine the criteria for existing diagnostic categories. This classification not only facilitates the diagnosis, treatment, and prognosis of AML. but also supports the design of innovative clinical trials, so several drugs are approved for patients with newly diagnosed AML, including G-CLAM (cladribine, cytarabine, filgrastim, and mitoxantrone), FLAG-IDA (fludarabine, cytarabine, idarubicin, and filgrastim), and lomustine. However, the cornerstone of intensive chemotherapy remains anthracycline- and cytarabine-based regimens [4]. The estimated 5-year overall survival (OS) rate is 30%, though this rate varies across different age groups. It reaches 35% to 50% in younger patients, but drops to less than 10% in patients over 60 years of age [4]. At 10 years, the survival rate declines further to 15% for those under 60 years and to 2% for those over 60 [5]. These data reveal that, despite advancements in treatment strategies, including immunotherapy and targeted therapy, relapses in AML patients remain fatal, with chemoresistance and relapse being the primary causes of AML-related deaths [1,6,7]. Given the absence of a definitive curative therapy, some authors advocate for active participation in clinical trials as a highly recommended course of action [8]. Relapses or refractory disease result from a lack of sensitivity to chemotherapy [6,9], making the overcoming of chemoresistance a current challenge.

Chemoresistance to conventional treatments such as cytarabine (Ara-C) and daunorubicin is a main cause of relapse, recurrence, metastasis, and high mortality in AML patients [10]. Therefore, reversing Ara-C resistance is indispensable for the improvement of clinical outcomes [11]. For Ara-C to function as an antileukemic agent, it must first be transported into the cell either by simple diffusion or via membrane transporters such as human equilibrium nucleoside transporter 1 (hENT1). Once inside the cell, cytarabine undergoes sequential phosphorylation by deoxycytidine kinase (dCK), deoxycytidine monophosphate kinase (dCMPK), Ara-C diphosphate (Ara-CDP), and nucleoside diphosphate kinase (NDPK), to produce the active metabolite Ara-C triphosphate (Ara-CTP). This metabolite is then incorporated into DNA during synthesis, where it blocks cell cycle progression and ultimately induces apoptosis [12,13].

It is well established in the literature that cytarabine resistance can arise from several mechanisms, including low expression of the hENT1 transporter [14], diminished levels of deoxycytidine kinase (dCK) activity [6], and increased levels of inactivating enzymes such as CDA and SAMHD1 [15]. These factors compete with Ara-CTP incorporation into DNA, thereby preventing the production of its active form [16]. This reduction in the expression of ENT1 and dCK is a clinical feature of Ara-C resistance observed in AML patients and cell lines [15,16,17,18,19,20]. Notably, among these molecular entities, both MDR1 and MRP1 are more prevalent in older patients and in those with refractory, relapsed, or secondary disease, compared to patients with de novo AML [16]. Since cytarabine is used in the induction, consolidation, and maintenance therapy of AML treatment [6,9], the development of chemoresistant leukemic lines enables the study of compounds that can overcome chemoresistance [21].

The use of natural products in cancer therapy is an active area of research, and few studies have been conducted to examine the anti-cancer effects of AML in vitro and in vivo using milk proteins [22]. Casein and its derivatives can attenuate tumor growth in different types of cancer [23]. In the context of leukemia, sodium caseinate (SC), a salt derived from casein, a milk protein, inhibits proliferation and induces apoptosis in WEHI-3, J774, or P388 mouse leukemic cell lines [23,24,25,26], but not in normal hematopoietic cells [23,24,27], and prolongs the survival of WEHI-3 tumor-bearing mice [23], thus demonstrating inhibitory effects on the proliferation of leukemia cells without damaging normal cells [23,24,25]. However, whether the SC effects are maintained in a cytarabine-resistant line remains unknown. In this context, our study aimed to evaluate the antileukemic potential of sodium caseinate in a cytarabine-resistant AML cell line, designed to mimic the chemoresistance observed in patients who fail to respond to further chemotherapy due to acquired resistance [6]. We demonstrate that SC inhibits the proliferation and induces apoptosis in cytarabine-resistant WEHI-CR50 cells. Moreover, SC increased survival in WEHI-CR50 tumor-bearing mice.

## 2. Materials and Methods

### 2.1. Cell Culture

The WEHI-3 mouse acute myeloid leukemia cells obtained from the American Type Culture Collection TIB-68 (ATCC TIB-68) were cultured in IMDM (Iscove’s Modified Dulbecco’s Medium Gibco BRL, Grand Island, NY, USA) and supplemented with 10% fetal bovine serum (FBS) (Gibco BRL, USA) and 1.1 µM β-mercaptoethanol (Gibco BRL, USA). The cells were cultured at a density of 1 × 10^4^ cells/mL under conditions of 37 °C and 5% CO_2_, and reseeding was performed every 48 h.

### 2.2. Development of the Cytarabine-Resistant Subline

The parental WEHI-3 cell line (2 × 10^4^ cell/mL) was exposed to increasing concentrations of cytarabine, as described in Lambarry 2020 [28], at a concentration of 197 nM. Each increase in the drug concentration was carried out when the proliferation was similar to that of the parental cells in the absence of cytarabine; thus, this subline was named WEHI-CR25. Following resistance induction, the dose of cytarabine for WEHI-CR25 cells was increased until it reached a concentration of 443.25 nM, a concentration similar to the IC50 of parental WEHI-3 cells. This adjustment ensured that the proliferation and viability of the WEHI-CR25 cells were similar to those of the sensitive cells, resulting in the designation of the modified cell line as WEHI-CR50.

### 2.3. Cell Proliferation and Viability Curves

Viability was determined by using trypan blue exclusion assays (Sigma, St. Louis, MO, USA) in a Neubauer chamber. Unstained cells were considered viable, while those stained blue were considered dead cells, indicating the percentage of viable cells.

Parental WEHI-3 cells were cultured in quadruplicate for 72 h in 96-well plates at a density of 2 × 10^4^/mL with or without the addition of different concentrations of cytarabine (0, 123, 184, 245.5, 307, 368, 429.5, 497, 593, 654.5, or 716 nM). Likewise, the WEHI-CR25 and WEHI-CR50 cell lines were grown in the presence of increasing concentrations of cytarabine (0 to 17,440.4 nM and 0 to 6785.3 nM, respectively). All the samples were subjected to independent tests in triplicate. The MTS technique (CellTiter 96^®^ AQueousOne Solution Cell Proliferation Assay, Madison, WI, USA) was used to evaluate cell proliferation after growth, and absorbance levels were determined using a plate reader (Multiskan Go, Waltham, MA, USA). The above data were used to calculate the IC50 values for parental WEHI-3, WEHI-CR25, and WEHI-CR50 cells.

### 2.4. Reverse Transcriptase–Polymerase Chain Reaction

The expression of the genes of interest with respect to chemoresistance was determined in WEHI-CR25 and WEHI-CR50 cells by real-time RT-PCR. Total RNA was isolated using the RNeasy Mini Kit 250 (Qiagen, Hilden, Germany), according to the manufacturer’s instructions. Total RNA concentration and purity were evaluated using a NanoDrop spectrophotometer (ThermoScientific, Wilmington, DE, USA). Relative mRNA expression of ENT1, dCK, MDR1 and MRP1 was determined and related to the expression of the housekeeping gene β-actin, using the 2^^ΔΔ^Cq method. Gene expression was determined using SYBR Green system in an Applied Biosystems USA thermocycler with the corresponding primer sets given in Table 1. The RT reaction mixture was incubated for 10 min at 50 °C, followed by 5 min at 95 °C, 40 cycles of denaturation at 95 °C for 10 s, annealing at 60 °C for 30 s, and extension at 72 °C for 15 s. All the samples were subjected to independent tests in triplicate.

### 2.5. Immunofluorescence

WEHI-CR50 cells were fixed with 4% paraformaldehyde and then permeabilized using 0.25% Triton X-100 for 15 min. After permeabilization, the cells were rinsed with PBS and blocked with 1% bovine serum albumin (Sigma-Aldrich, St. Louis, MO, USA) for 1 h. For staining, cells were incubated overnight at 4 °C with the following primary antibodies: Alexa Fluor^®^488 mouse anti-dCK (1:100 dilution, Santa Cruz, catalog no. sc-393099) and Alexa Fluor^®^594 mouse anti-ENT1 (1:100 dilution, Santa Cruz, catalog no. sc-377283). Following the overnight incubation, the cells were rinsed three times with PBS. To visualize the nuclei, the cells were counterstained with 6-diamidino-2-phenylindole (DAPI, Burlingame, CA, USA). Immunofluorescence was evaluated using a confocal inverted microscope (TCS-SP2, Leica, Heidelberg, Germany). Each experiment was conducted in triplicate across different groups, to ensure reproducibility.

### 2.6. In Vitro SC Assays

Sodium caseinate (Spectrum, New Brunswick, NJ, USA) was dissolved in PBS, and autoclaved dilutions were made with PBS to achieve concentrations of 0, 2, 4, and 8 mg/mL SC. Parental WEHI-3, WEHI-CR25, and WEHI-CR50 cells were cultured in 96-well plates (Corning Costar, St. Louis, MO, USA) as described above, and then stimulated with SC. The crystal violet technique was used to evaluate cell proliferation. The optical density at 570 nm was read, and the absorbance was determined using a plate reader (Multiskan Go, USA).

### 2.7. Apoptosis Assay

An apoptosis assay was performed using a PE Annexin V Apoptosis Detection Kit I (BD Biosciences, San José, CA, USA), according to the manufacturer’s instructions. Briefly, 2 × 10^4^/mL WEHI-CR50 cells were incubated with SC or vehicle (PBS) for 72 h. All adherent and floating cells were harvested and then suspended in 1× binding buffer at 1 × 10^5^ cells/mL. This cell suspension was transferred to an Annexin V-Phycoerythrin (PE) conjugate solution, and 7-amino actinomycin D (7-AAD) was added. The cells were incubated in the dark for 15 min at room temperature. The fluorescence of Annexin V-PE and 7-AAD (at 528 nm and 650 nm, respectively) was analyzed using a BD FACSAria II flow cytometer (BD Biosciences, San Jose, CA, USA).

### 2.8. Survival Curves

Male BALB/c mice, aged 4 months, were housed under pathogen-free conditions in 12 h of light and 12 h of darkness, at controlled temperatures, and were fed a standard laboratory diet. A total of forty healthy mice were randomly divided into eight groups, each with five mice. Group I consisted of healthy mice that did not receive leukemic cells. Groups II, III, and IV were mice injected intraperitoneally (i.p.) with leukemic parental WEHI-3 cells. These groups included untreated controls, PBS-treated (vehicle), or 2 g/kg SC-treated leukemic mice, respectively. Groups V, VI, VII, and VIII consisted of mice injected i.p. with WEHI-CR50 cells. These groups included untreated controls, PBS-treated, 2 g/kg SC-treated, or 4 g/kg SC-treated leukemic mice, respectively. Parental WEHI-3 or WEHI-CR50 cells were washed twice with PBS, counted using trypan blue to confirm >95% viability, and adjusted to a concentration of 2.5 × 10^5^ cells/mL. SC treatment was initiated 24 h after cell injection and administered every 48 h for 41 days. Mice were monitored for survival every 24 h. The following behavioral or postural changes were considered endpoints: reduced activity, hunched posture, abdominal swelling, and difficulty breathing. If three of these symptoms appeared in two consecutive examinations, they were deemed reliable indicators of imminent death. Euthanasia was performed by CO_2_ inhalation followed by cervical dislocation (NOM-062-ZOO-1999) [29]. Careful handling techniques were employed to minimize stress during all procedures, and postmortem examinations were conducted to confirm euthanasia.

All animal procedures were reviewed and approved by the Bioethics and Security Committee of the Faculty of High Studies, Zaragoza, in compliance with national and international regulations for animal welfare (FESZ/DEPI/CI/216/14). The procedures adhered to the Official Mexican Standard NOM-062-ZOO-1999 and the guidelines established for research and evaluation methodologies or traditional medicine (WHO, 2002). Survival was evaluated using Kaplan–Meier analysis, and survival curves between groups were compared using the log-rank test. All statistical analyses were performed using GraphPad Prism 6 software (San Diego, CA, USA).

### 2.9. Statistical Analysis

The experiments were repeated three times, and one-way ANOVA followed by Tukey’s test was used for statistical analysis. We used the GraphPad Prism 8 software. Statistical significance is indicated as the following: * *p* < 0.05, ** *p* < 0.01, *** *p* < 0.001, **** *p* < 0.0001. ns, non-significant. All values are means ± DS. For survival analysis, the nonparametric Wilcoxon test was used. *p* < 0.05 was considered to indicate a statistically significant difference. Statistical SPSS software 25 (SPSS, Inc., Chicago, IL, USA).) was used to perform the analyses.

## 3. Results

### 3.1. Sensitivity of Parental WEHI-3 Cells to Cytarabine

**Figure 1:**
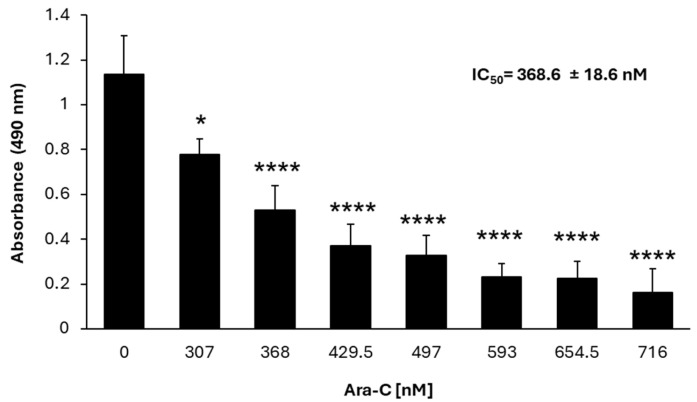
Cytarabine (Ara-C) inhibits the proliferation of parental WEHI-3 cells in a dose-dependent manner. The proliferation of 2 × 10^4^/mL parental WEHI-3 cells, treated with different of cytarabine (Ara-C) for 72 h was evaluated using the MTS assay. Data are presented as means ± SDs from three independent experiments performed in quadruplicate. Statistical significance was determined using one-way ANOVA followed by Tukey’s post hoc test. Significance levels are indicated as * *p* < 0.05, **** *p* < 0.0001.

To test the sensitivity of parental WEHI-3 cells to cytarabine, a chemotherapeutic agent commonly used in the treatment of leukemia was employed. A proliferation curve for the parental WEHI-3 cells was generated in the presence of increasing concentrations of cytarabine. The results indicated a concentration-dependent inhibition of proliferation, with a calculated IC50 of 368.6 nM for cytarabine (Figure 1).

### 3.2. Development of Cytarabine-Resistant Cells

**Figure 2:**
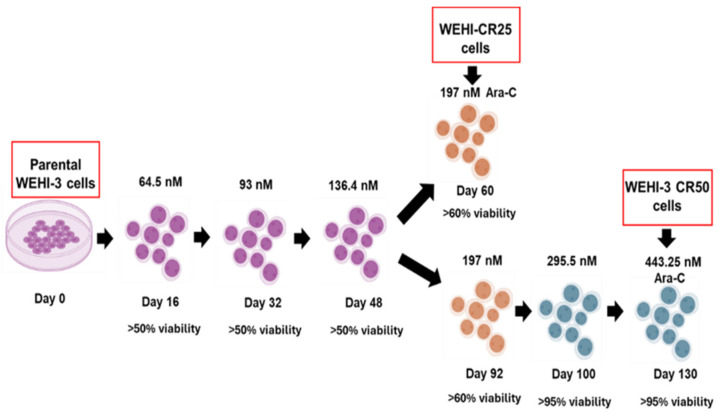
Workflow for generating the WEHI-CR25 and WEHI-CR50 cell sublines resistant to Ara-C. This figure illustrates the process for developing cytarabine-resistant sublines, derived from the parental WEHI-3 cells.

To develop cytarabine-resistant sublines, a model was established using parental WEHI-3 cells. Some authors suggest that resistant can be considered successful when proliferation remains consistent at supra-IC50 concentrations [30]. Thus, the cell culture began with 64.5 nM cytarabine and progressed to concentrations of 197 nM and 443.25 nM cytarabine to obtain the WEHI-CR25 and WEHI-CR50 cells, respectively (Figure 2).

### 3.3. Cytarabine Inhibits the Proliferation of Parental WEHI-3 Cells but Does Not Affect the Proliferation of WEHI-CR25 and WEHI-CR50 Cells

**Figure 3:**
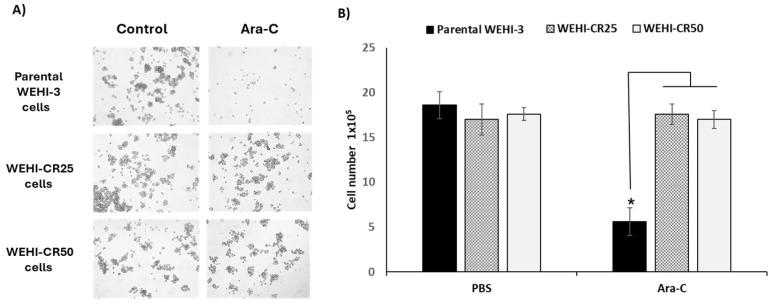
Effect of cytarabine on parental WEHI-3, WEHI-CR25 and WEHI-CR50 cells. (**A**) Photographs from a representative experiment showing parental WEHI-3, WEHI-CR25 and WEHI-CR50 cells in the absence or presence of cytarabine. (**B**) Number of cells in the parental and resistant sublines treated with PBS or cytarabine (Ara-C, 197 nM). The initial cellular density for all conditions was 2 × 10^4^/mL. Data are presented as means ± SDs from three independent experiments conducted in quadruplicate. Statistical significance was determined using one-way ANOVA followed by Tukey’s post hoc test, with * *p* < 0.05 indicating significance.

To evaluate cytarabine sensitivity, parental WEHI-3, WEHI-CR25 and WEHI-CR50 cells were incubated with 368.6 nM cytarabine for 72 h. Interestingly, we observed specifically a decrease in the proliferation of the parental WEHI-3 cells compared to the WEHI-CR25 and WEHI-CR50 sublines (Figure 3A,B). These results suggest the generation of two cytarabine-resistant sublines by not altering the cell number. With this evidence of resistance, the WEHI-CR25- and WEHI-CR50-resistant cells were subsequently maintained with 197 nM and 443.25 nM cytarabine, respectively.

### 3.4. The Resistance of the WEHI-CR25 and WEHI-CR50 Cells to Cytarabine Modulates the Expression of Genes Involved in Chemoresistance

**Figure 4:**
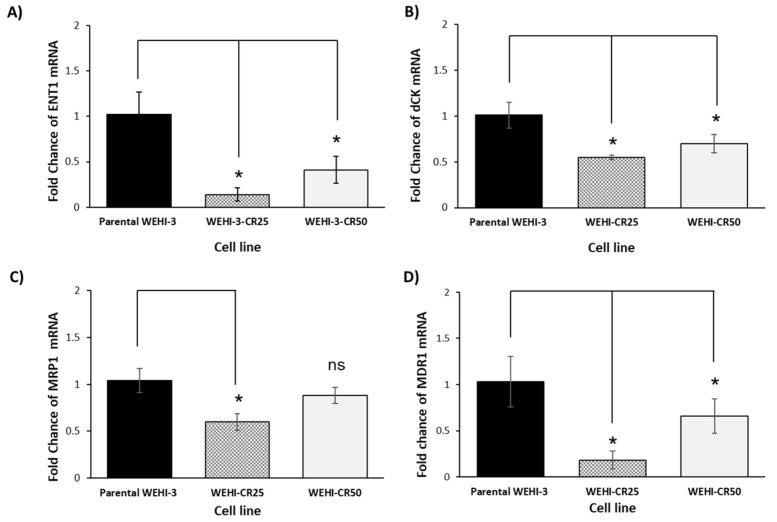
Expression of genes related to cytarabine resistance in the WEHI-CR25 and WEHI-CR50 cells. (**A**) Real-time RT-PCR expression analysis of ENT1. (**B**) dCK. (**C**) MDR1 and (**D**) MRP1 (all data represent the Fold-Change mean value of mRNA expression of three independent measurements). The results were analyzed using one-way ANOVA followed by Tukey’s post hoc test (* *p* < 0.05). Abbreviations: ENT1, equilibrium nucleoside transporter 1; dCK, deoxycytidine kinase; MRP1, multidrug resistance protein 1; MDR1, multidrug resistance gene 1; ns, not significant.

To evaluate whether cytarabine-resistant sublines modulate the expression of genes involved in resistance to cytarabine, a real-time RT-PCR assay was performed. We observed that the levels of both ENT1 and dCK were significantly lower in the resistant cell lines compared to the parental WEHI-3 cells (Figure 4A,B). Additionally, evaluation of other genes related to multidrug resistance, such as MRP1 and MDR1, revealed that only the level of MDR1 was significantly reduced in both cytarabine-resistant lines compared to the parental WEHI-3 cells (Figure 4C,D). These results suggest that the generation of these two cytarabine-resistant sublines was associated with a decrease in the expression of genes involved in chemoresistance.

### 3.5. Sodium Caseinate Inhibits the Proliferation of WEHI-CR25 and WEHI-CR50 Cells, Similarly to Parental WEHI-3 Cells

**Figure 5:**
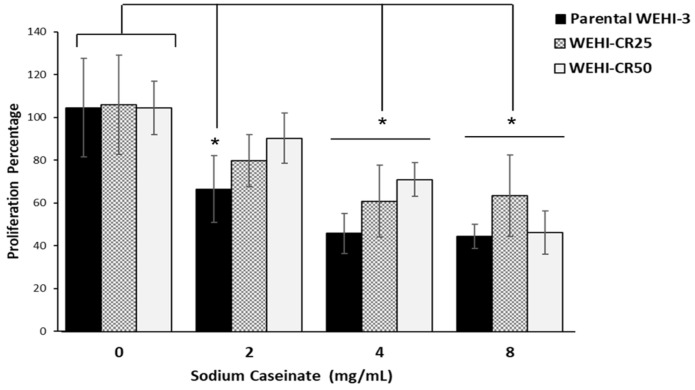
Antiproliferative effect of sodium caseinate on parental WEHI-3, WEHI-CR25, and WEHI-CR50 cells. Proliferation curves of parental WEHI-3 cells and cytarabine-resistant WEHI-CR25 and WEHI-CR50 cells exposed to increasing concentrations of sodium caseinate, as assessed by the crystal violet staining technique. Data are presented as means ± SDs from three independent experiments performed in quadruplicate. Statistical significance was determined using two-way ANOVA followed by Tukey’s post hoc test with respect to the control groups (* *p* < 0.05).

Due to the anti-cancer potential of sodium caseinate in AML, we investigated whether WEHI-CR25 and WEHI-CR50 sublines remained sensitive to SC. We observed that SC at concentrations of 4 and 8 mg/mL significantly reduced the proliferation of both the WEHI-CR25 and WEHI-CR50 sublines, similar to the effect observed in the parental WEHI-3 cells (Figure 5). These result indicate the sensitivity of CS on the WEHI-CR50 subline.

### 3.6. Sodium Caseinate Induces Apoptosis and Modulates the Expression of Genes Associated with Resistance in WEHI-CR50 Cells

**Figure 6:**
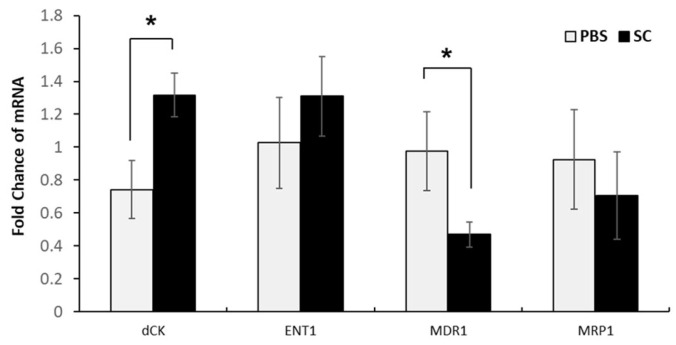
Expression levels of mRNAs related to cytarabine resistance in WEHI-CR50 cells treated with sodium caseinate. mRNA expression analysis of ENT1, dCK, MRP1, and MDR1 in WEHI-CR50 cells treated with sodium caseinate or phosphate-buffered saline (PBS) (negative control) for 72 h. β-actin was used as a housekeeping gene for quantification and normalization of the expression of resistance-associated genes, determined using the 2^^ΔΔ^Cq method. Data are presented as means ± SDs from three independent experiments. Statistical significance was assessed using Student’s *t*-test (* *p* < 0.05). Abbreviations: ENT1, equilibrium nucleoside transporter 1; dCK, deoxycytidine kinase; MRP1, multidrug resistance protein 1; MDR1, multidrug resistance gene 1.

**Figure 7:**
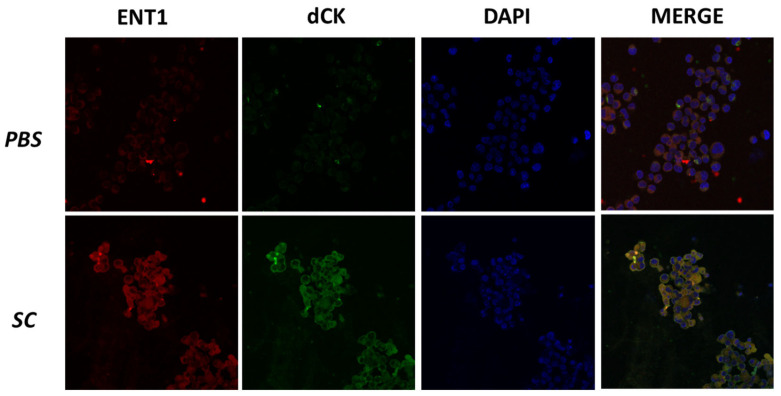
The treatment with SC regulates the expression of proteins related to cytarabine resistance. Representative images of confocal microscopy against ENT1 and dCK (**left panel**); nuclei stained with DAPI (**middle panel**); and merged images (**right panel**). Scale bar 50 µm. Abbreviations: ENT1, equilibrium nucleoside transporter 1; dCK, deoxycytidine kinase; DAPI, 4′,6-diamidino-2-phenylindole.

To determine whether the effect of SC on resistant cells extended beyond cell proliferation, we evaluated the expression of genes associated with chemoresistance using both qRT-PCR and immunofluorescence techniques. We observed that SC increased the expression of dCK and ENT1 (Figure 6 and Figure 7), but decreased MDR1, compared to the control group (Figure 6).

**Figure 8:**
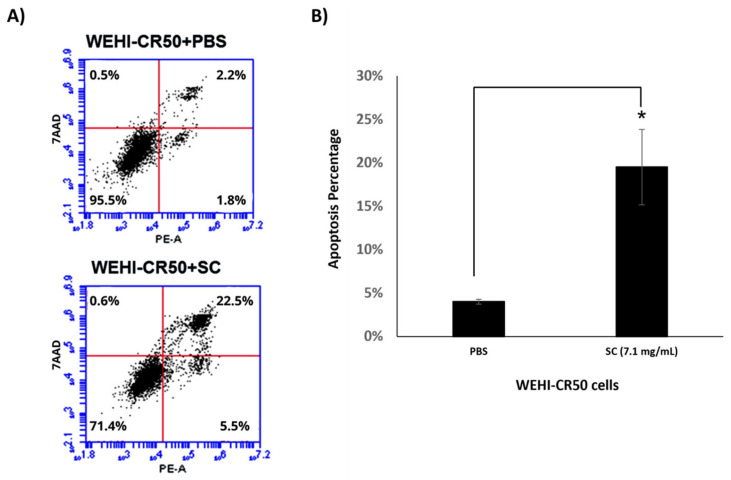
Apoptosis assay in WEHI-CR50 cells treated with sodium caseinate. (**A**) The distribution of WEHI-CR50 cells into early- and late-apoptosis groups, as well as the number of viable cells, was determined using flow cytometry with Annexin V-Phycoerythrin (PE) and 7-aminoactinomycin D (7-AAD) staining. (**B**) The percentage of total apoptosis (including both early and late stages) in WEHI-CR50 cells treated with sodium caseinate (SC) or phosphate-buffered saline (PBS) for 72 h. Data are presented as means ± SDs from three independent experiments. Statistical significance was assessed using Student’s *t*-test (* *p* < 0.05).

Additionally, we assessed phosphatidylserine translocation as an indicator of apoptosis. We found that WEHI-CR50 cells treated with SC (7.1 mg/mL) showed a significant 7-fold increase in the percentage of apoptotic cells, reaching 28% compared to the control group (Figure 8). Notably, these findings suggest that SC sensitizes the WEHI-CR50 cells, reversing chemoresistance and improving the therapeutic effect of Ara-C.

### 3.7. Sodium Caseinate Increases the Survival of Leukemic Mice

**Figure 9:**
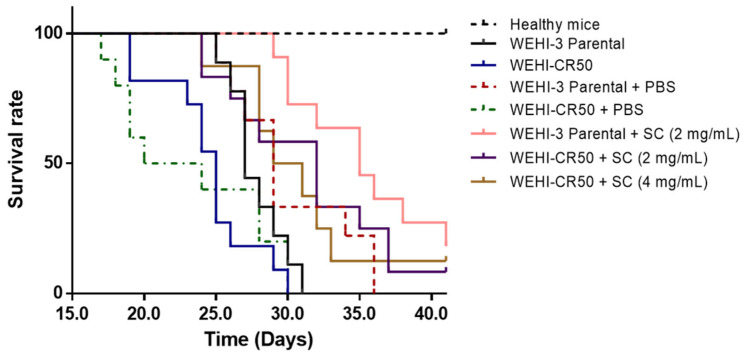
Effect of sodium caseinate on the survival of leukemic mice bearing parental WEHI-3 or WEHI-CR50 cells. Survival of BALB/c leukemic mice inoculated with either parental WEHI-3 or WEHI-CR50 cells and treated with 1 mL of vehicle (parental WEHI-3 + PBS) or SC at doses of 2 and 4 g/kg (parental WEHI-3 + SC 2 g/kg, WEHI-CR50 + SC 2 g/kg or WEHI-CR50 + SC 4 g/kg) administered every 48 h. Healthy mice (no WEHI cells), parental WEHI-3 mice (inoculated with WEHI-3), and WEHI-CR50 mice (inoculated with WEHI-CR50) served as controls. Survival curves were generated using Kaplan–Meier analysis. Statistical significance was assessed using the Wilcoxon test with comparisons with parental WEHI-3 + PBS and WEHI-CR50 + PBS.

To evaluate the effect of sodium caseinate (SC) in mice inoculated with either parental or cytarabine-resistant leukemia cells, we injected BALB/c mice intraperitoneally with parental WEHI-3 or WEHI-CR50 cells. The data revealed that 100% of the untreated leukemic mice succumbed to the disease, with maximum survival times of 31 days for mice inoculated with parental WEHI-3 cells and 30 days for those inoculated with WEHI-CR50 cells. Notably, all doses of SC significantly prolong the survival of mice inoculated with WEHI-CR50 cells in a manner similar to that observed in mice inoculated with parental WEHI-3 cells (Figure 9).

## 4. Discussion

Chemoresistance is a severe problem in the treatment of AML, in which cytarabine is used for remission induction, consolidation, and maintenance therapy [31,32]. Developing a model of resistance to this drug is of great value in oncology. It provides an approach to investigate the mechanisms of cytotoxicity and resistance to chemotherapeutic agents [18,31,32]. To this end, we generated two leukemia cell sublines resistant to cytarabine. Notably, the WEHI-CR25 and WEHI-CR50 cell lines proliferate at cytarabine concentrations close to the IC50 of the parental WEHI-3 cells, a requirement that some authors recommend for achieving chemoresistance [33,34,35]. Our results are consistent with these studies, showing a significant decrease in the proliferation of the parental WEHI-3 cells compared to the WEHI-CR25 and WEHI-CR50 cells (Figure 3). These results suggest that the generation of these two cytarabine-resistant sublines did not alter the overall proliferation rate of cell proliferation.

The primary mechanism underlying cytarabine resistance appears to involve the intracellular levels of the active metabolite Ara-CTP, which may result from low levels of the ENT1 transporter and dCK, an activating enzyme [6,19,21]. When we analyzed the expression of these two genes, which are associated with cytarabine resistance in WEHI-CR25 and WEHI-CR50 cells, we found that their expression levels were reduced, compared to those in the parental WEHI-3 cells. This negative modulation of ENT1 and dCK gene expression aligns with findings in the cytarabine-resistant HL-60 human leukemic cell line and in chemoresistant acute myeloid leukemia samples [31,36]. In contrast, AML in elderly patients is more likely to be resistant to treatment, due to the overexpression of MDR1 and MRP1, two genes involved in the expulsion of chemotherapeutic agents [32,37] as observed in HL-60 and K562 cell lines resistant to cytarabine [38]. However, in our study, we found that the expression of both genes was lower in WEHI-CR25 and WEHI-CR50 cells than in the parental WEHI-3 cell line. Similarly, the human myeloid leukemia cell line KF-19VCR lacks expression of the MDR1 gene and P-glycoprotein, and does not exhibit alterations in deoxycytidine kinase and deaminase activities, yet it remains resistant to cytarabine. Moreover, MDR1, MRP1, and LRP/MVP were not associated with clinically resistant disease in AML samples from patients at diagnosis or in those with relapsed or refractory disease [39,40]. The transformation of AML cells from a sensitive state to one resistant to treatment after exposure to a chemotherapeutic agent can be explained by, but not limited to, an increased ability of leukemic cells to export pharmacologically active agents and a decrease in the ratio of active agents due to changes in the expression and function of enzymes responsible for drug activation. However, drug resistance can also be attributed to several other molecular mechanisms, including increased DNA damage repair, decreased expression or function of pro-apoptotic factors, upregulation of anti-apoptotic genes, enhanced stress responses, and even aberrant oxygen metabolism [10,32,40]. Our findings in WEHI-CR25 and WEHI-CR50 cells thus support the controversial role of MDR1 and MRP1 expression in cytarabine-resistant acute myeloid leukemia. The next challenge is to determine whether another molecular mechanism is involved in the increase in resistance to cytarabine in the WEHI-CR25 and WEHI-CR50 cell lines.

McDermott et al. [35] categorized drug-resistant cell models into two groups: clinically relevant models and high-level laboratory models. In the former, resistance is defined by a two- to eightfold increase in the IC50 value, compared to that of the parent cell line, aiming to replicate the conditions experienced by cancer patients during chemotherapy. In contrast, high-level laboratory models involve the use of high drug doses, with treatment doses often escalated over time. These models are primarily utilized to investigate molecular changes associated with resistance, though they are less applicable as clinical models [30]. The 6-fold increase in resistance to cytarabine for WEHI-CR25 was barely in the resistance range corresponding to the clinical relevance model. The 16-fold increase in resistance to cytarabine observed in WEHI-CR50 cells categorizes this model as a high-level laboratory model in which genetic alterations are more stable and easier to detect; therefore, this model is relevant for studying the mechanisms of chemoresistance and chemosensitization [30]. Given these findings, we exposed WEHI-CR50 cells to the same doses of SC that have previously been shown to reduce proliferation and induce apoptosis in parental WEHI-3 cells [23,24]. Surprisingly, SC continued to suppress cell proliferation and induce apoptosis in the resistant cell line. Our results showed that the reduction in proliferation and induction of apoptosis in cytarabine-resistant cells were linked to increased expression of ENT1 and dCK, along with decreased expression of MDR1. However, the specific mechanism of action of SC remains unclear. Several hypotheses have been proposed regarding how caseins, the primary component of SC, might modulate the proliferation, viability, and differentiation of both normal hematopoietic and leukemic cells [22]. These hypotheses include the involvement of Toll-like and opioid receptors, as well as the induction of opioid peptides and proinflammatory cytokines [27,41,42]. Nonetheless, further research is necessary to elucidate the precise mechanism of action of SC.

The downregulation of deoxycytidine kinase (dCK) is recognized as one of the key molecular events driving the development of cytarabine resistance [22,23,43,44]. Notably, WEHI-CR50 cells exhibit significantly reduced dCK expression. Remarkably, treatment with SC led to an increase in ENT1 and dCK expression, suggesting that SC not only reduces proliferation and induces apoptosis in resistant cells, but may also sensitize these cells to cytarabine, thereby enhancing the drug’s effectiveness. Additionally, SC treatment resulted in a further reduction in MDR1 expression, which was already lower in WEHI-CR50 cells compared to parental WEHI-3 cells. This decrease in MDR1 expression likely impairs the ability of these cells to export pharmacologically active agents, potentially increasing their sensitivity to cytarabine.

Previous studies have shown that casein and its derivatives can attenuate tumor growth in different types of cancer, including leukemia [22,26,43,44,45]. This beneficial effect occurs through (a) the suppression of stem cell markers (such as CD44) [22] and cell adhesion molecules like uPAR/PAI-1, which are involved in tumor progression and metastasis, (b) the cell cycle inhibition [46], and (c) the increase in cell death by apoptosis [22,23,47,48]. Our research group has previously described a potential molecular mechanism of casein and its derivatives (sodium caseinate), for cancer therapy, including leukemia [22]. These mechanisms involve multiple signaling pathways, including the following: the activation of interferon-associated STAT1 signaling, the suppression of stemness-related markers such as CD44, the inhibition of STAT3/HIF1-α signaling, the downregulation of uPAR and PAI-1 expression, the loss of mitochondrial membrane potential and subsequent reduction in intracellular ATP production, the activation of caspase-3, and the suppression of TLR4/NF-κB signaling. In the current study, survival was evaluated in mice with leukemia induced by cytarabine-resistant WEHI-3 cells. Specifically, we induced leukemia in BALB/c mice using WEHI-CR50 cells and administered the same dose of SC (2 mg/kg) that had been effective in the parental cell model [23,24]. Additionally, we tested whether higher doses of SC could further prolong the survival of leukemic mice, as the effectiveness of cancer therapy is often dose-dependent. The data demonstrated that SC significantly prolonged survival in both models, those using parental WEHI-3 cells and those using resistant WEHI-CR50 cells, regardless of the dose. Increasing the dose from 2 to 4 mg/kg had a marginal and nonsignificant effect on survival. Previous reports indicated that SC reduces the proliferation of WEHI-3 cells in vitro and prolongs the survival of leukemic mice in vivo [23]. Our findings confirm that both the clinically relevant model and the high-level laboratory model are sensitive to SC, similar to the parental WEHI-3 cell line.

## 5. Conclusions

The therapeutic strategy of utilizing cytarabine-resistant AML sublines aims to model relapse and resistance to anticancer agents, which are significant causes of mortality in patients. Despite their resistance, these sublines remain sensitive to sodium caseinate, a milk protein, both in vitro and in vivo, similar to the parental WEHI-3 cells. Our findings demonstrate that SC is a potent anti-tumor agent that not only reduces proliferation and induces apoptosis in resistant cells, but may also enhance the effectiveness of cytarabine by increasing the expression of ENT1 (equilibrium nucleoside transporter 1) and dCK (deoxycytidine kinase). Additionally, SC prolongs the survival of WEHI-CR50 tumor-bearing mice in a manner similar to its effects on mice inoculated with parental WEHI-3 cells. These results suggest that further investigation into the use of natural products, particularly the combination of SC with cytarabine, is necessary for improving AML treatment.

## Figures and Tables

**Table 1:** Sequence of primers.

Gen	Forward Sequences (5′→3′)	Reverse Sequences (5′→3′)
ENT1	5′-CTGGAAAGGCGTAGAGGCTG-3′	5′-CTTCCCTTCGCAGACTGCTT-3′
dCK	5′-AGCAGTGAGTCTGGAGGTAG-3′	5′-GAGAAGGCAGAGAAGGCTGG-3′
SIRT1	5′-CGGCTACCGAGGTCCATATAC-3′	5′-CAGCTCAGGTGGAGGAATTGT-3′
MDR1	5′-GTGGTGTCATTGTGGAGCAAG-3′	5′-GCATCAGTGTCACTCTGGGATC-3′
MRP1	5′-CAGTGGTTCAGGGAAGGATTTA-3′	5′-CACTGTGGGAAGACGAGTTGCT-3′
β-actin	5′-CACTGTCGAGTCGCGTCC-3′	5′-CGCAGCGATATCGTCATCCA-3′

## Data Availability

Data are unavailable, due to privacy.
